# Magnetization Reversal by Out-of-plane Voltage in BiFeO_3_-based Multiferroic Heterostructures

**DOI:** 10.1038/srep10459

**Published:** 2015-05-21

**Authors:** J. J. Wang, J.M. Hu, Ren-Ci Peng, Y. Gao, Y. Shen, L. Q. Chen, C. W. Nan

**Affiliations:** 1 State Key Lab of New Ceramics and Fine Processing, School of Materials Science and Engineering, Tsinghua University, Beijing, 100084, China; 2 Department of Materials Science and Engineering, The Pennsylvania State University, University Park, Pennsylvania, 16802, USA

## Abstract

Voltage controlled 180° magnetization reversal has been achieved in BiFeO3-based multiferroic heterostructures, which is promising for the future development of low-power spintronic devices. However, all existing reports involve the use of an in-plane voltage that is unfavorable for practical device applications. Here, we investigate, using phase-field simulations, the out-of-plane (i.e., perpendicular to heterostructures) voltage controlled magnetism in heterostructures consisting of CoFe nanodots and (110) BiFeO_3_ thin film or island. It is predicted that the in-plane component of the canted magnetic moment at the CoFe/BiFeO_3_ interface can be reversed repeatedly by applying a perpendicular voltage across the bottom (110) BiFeO_3_ thin film, which further leads to an in-plane magnetization reversal in the overlaying CoFe nanodot. The non-volatility of such perpendicular voltage controlled magnetization reversal can be achieved by etching the continuous BiFeO_3_ film into isolated nanoislands with the same in-plane sizes as the CoFe nanodot. The findings would provide general guidelines for future experimental and engineering efforts on developing the electric-field controlled spintronic devices with BiFeO_3_-based multiferroic heterostructures.

Multiferroic magnetoelectric (ME) heterostructures are comprised of at least two different ferroic (ferromagnetic, ferroelectric, antiferromagnetic, etc.) materials that are artificially combined with well-defined interface^1^,^2^,^3^,^4^,^5^. In multiferroic ME heterostructures, the ability to switch magnetization directly using an electric voltage rather than electric current (i.e., magnetoelectric coupling) can potentially be implemented to solve the overheating problem in spintronic devices^6^,^7^,^8^. Among the various multiferroic ME heteostructures, the magnet/BiFeO_3_ (BFO) heterostructure is attracting increasing research efforts^9^,^10^,^11^,^12^,^13^,^14^,^15^,^16^,^17^,^18^, in which the ferroelectric (FE) polarization **P** is coupled to the magnetization **M** in the overlaying magnet, based on the intrinsic room-temperature ME coupling between **P** and the antiferromagnetic axis **L** in the BFO, and the exchange coupling between **M** and **L**. In particular, in Co_90_Fe_10_ (abbreviated to CoFe)/BFO thin-film heterostructures, voltage controlled net magnetization reversal has been demonstrated driven by this interfacial exchange interaction^10^,^13^. A voltage controlled uniform (i.e., magnetic single-domain) magnetization reversal is further predicted under the action of both exchange interaction and ferroelastic strain^16^. However, in these CoFe/BFO heterostructures, the voltage was applied through in-plane electrodes grown on top of the BFO film^10^,^13^. These in-plane electrode configuration introduces unfavorable factors including too large coercive voltage, too large size limitation, and inconvenient integration into conventional microelectronics circuits. Therefore, an out-of-plane (i.e., perpendicular) voltage configuration across the BFO film is highly desired, for the design of high-performance spintronic devices^19^,^20^. Soon after we submitted the present article, a wonderful experimental work on the magnetization reversal by out-of-plane voltage in the CoFe/(110) BFO heterostructure was reported^21^, further indicating this future research tendency in the BFO-based multiferroic heterostructures. In this article, we demonstrate, using phase field method^16^,^22^,^23^, a mesoscale morphological engineering approach to achieving perpendicular voltage controlled magnetization reversal in magnetic/BFO thin-film heterostructures.

Consider the widely investigated Co_90_Fe_10_ (abbreviated to CoFe herein)/BFO heterostructure as an example. Compared to the (001)-oriented BFO films in previous CoFe/BFO heterostructures^10^,^13^,^16^, a (110)-oriented BFO film is utilized herein, which can be grown on a (110) SrTiO_3_ substrate with SrRuO_3_ as the bottom electrode^24^. An electric voltage is then applied perpendicularly through the SrRuO_3_ and the top CoFe dot that can be cut out from a continuous CoFe film by focused ion beams^25^, as shown in Fig. 1a. In order to demonstrate the perpendicular voltage-induced magnetization reversal, a phase field model is developed to understand and predict the switching behaviors of the polarization and magnetization in the (110) BFO thin film/island and the CoFe dot (see Methods).

## Results

### Principles of the Perpendicular Voltage Controlled Magnetization Reversal

**Figure 1:**
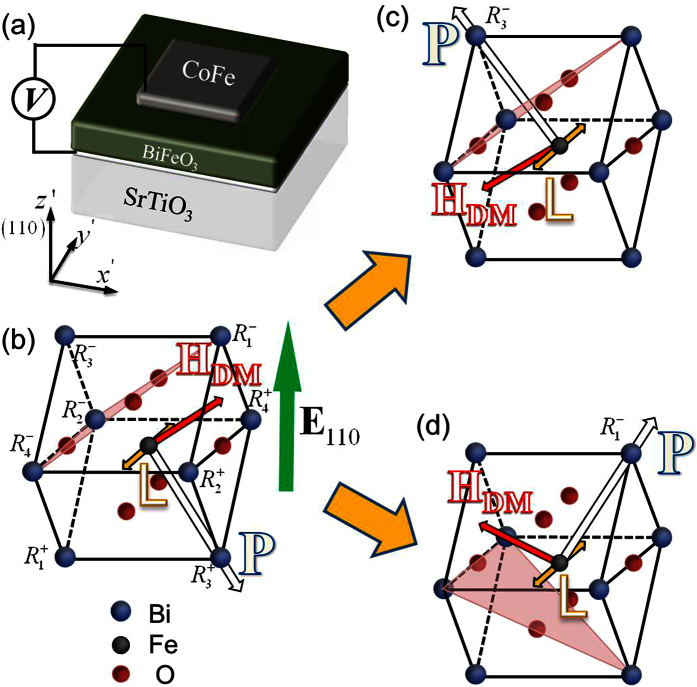
Voltage-induced ferroelectric and antiferromagnetic switching in CoFe/(110) BiFeO_3_ heterostructure. **** (**a**) Schematic illustration of the proposed CoFe/(110) BiFeO_3_ multiferroic heterostructure under perpendicular voltage modulation. (**b**) The lattice structures of (110) BiFeO_3_ film in 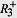 phase, and under perpendicular electric field along [110] direction, the polarization may switch by (**c**) 180° to 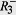 phase, or (**d**) 109° to 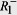 phase.

By engineering the substrate^26^,^27^ or pre-poling^24^ the (110) BFO film using piezoelectric force microscopy (PFM) before sputtering the CoFe layer, remnant polarization distributions with single-domain state or large surface-area (usually larger than 3 μm × 3 μm) individual domains can be obtained according to experiments^24^,^26^,^27^. Therefore, it is very likely that the patterned nanoscale CoFe dot lies on an individual domain of BFO with much larger size over microns, for instance, the 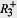 domain with **P** along 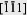 as shown in Fig. 1a. Given that the polarization **P**, the antiferromagnetic axis **L**, and the canted magnetic moment **M**_cant_ induced via the Dzyaloshinskii-Moriya (DM) interaction^28^,^29^ (the contribution of which can be described by an effective magnetic field **H**_DM_ along the same direction) are orthogonal to each other^30^,^31^ at the CoFe/BFO interface, i.e., **M**_cant_ (**H**_DM_) = **P** × **L**^13^,^32^, and also given that **L** is along the 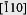 direction for the 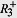 (i.e., **P**→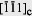) domain according to experimental observations^33^, the **H**_DM_ field should be along the 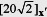 direction. Under a negative perpendicular voltage applied along the [110] direction between the SrRuO_3_ and CoFe electrodes, the polarization may either switch by 180° from 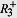 to 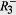 (i.e., **P**→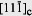) (Fig. 1c) or by 109° from 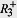 to 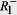
( i.e., **P**→[111]_c_) (Fig. 1d). Experiments also show that the antiferromagnetic axis **L** along 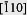 does not rotate^30^ for both switching paths, though the antiferromagnetic plane rotates during the 109° switching^33^. As a result, the **H**_DM_ field rotates from 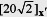 to 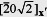 or 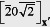 after the 109° or 180° switching, respectively. Such reversal of the in-plane component of **H**_DM_ field may further induce a reversal of in-plane net magnetization as demonstrated later. If the CoFe nanodot does not lie on an individual domain but on the domain wall of BFO, the electric field control of magnetization would be weakened because the interfacial exchange coupling can be neutralized due to the inhomogeneity of the polarization as well as the DM field at the BFO domain wall.

### Features of polarization/magnetization reversal in CoFe dot /(110) BFO film and island

**Figure 2:**
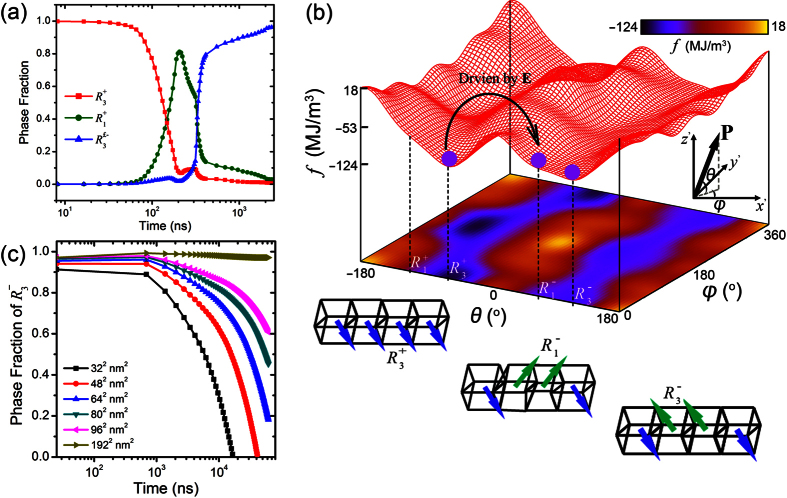
Mechanism of voltage-induced 180° ferroelectric switching in CoFe /(110) BiFeO_3_ heterostructure. **** For (110) BiFeO_3_ thin film with 80 × 80 nm^2^ CoFe dots grown above as the top electrode and ferromagnetic layer, the [110] electric field induced (**a**) phase evolutions and (**b**) the switching path 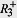 from first to 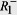 and then to 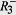, obtained from the total energy profile as a function of polarization orientation defined by *θ* and *ϕ* in inset schematic coordinate system. The total energy in (**b**) is illustrated by both the 3-Dimensional grid curves and 2-Dimensional color contour map. The inset lattice structures illustrate the polarization orientation of 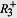, 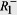 and 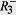 phase (i.e., **P**→
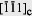
for 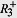, **P**→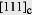 for 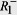, and **P**→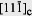 for 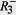, see Fig. 1). (**c**) Phase evolutions of 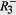 after removing the voltage for CoFe/(110)-film BiFeO_3_ multiferroic heterostructures with different CoFe size.

As shown in Fig. 2a, by applying a negative voltage (i.e., with upward electric fields) of −2*V*_c_ (*V*_c_ being the coercive voltage) across the (110) BFO film through the CoFe dot with an in-plane size of 80 nm × 80 nm and SrRuO_3_ bottom electrode, the volume fraction of 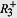 domain in the region underneath the CoFe drops dramatically after about 5  ns. Meanwhile, the volume fraction of 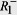 domain increases greatly and reaches a maximum value of 81% (with respect to the poled region) after about 200 ns and then decreases rapidly. This is accompanied by the dramatic increase in the volume fraction of 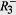 domain. After 1500 ns, the polarizations underneath the CoFe dot are stabilized almost as a 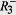 single-domain with a volume fraction of 96.3%. This sub-μs ferroelectric switching time is consistent with experimental observations in BiFeO_3_ and Pb(Zr,Ti)O_3_ films^34^,^35^. Such polarization reversal via successive 109° switching from 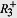 to 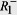 and 71° switching from 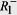 to 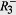 under perpendicular voltage has been experimentally observed in (110) and (001) BFO films^24^,^36^.

To understand the underlying physics for this ferroelectric switching path, we calculate the total free energy density profile by setting the polarization in the region underneath the CoFe dot of 192 nm × 192 nm pointing along every directions (see the orientation angles *θ* and *ϕ*) in the *x*’*y*’*z*’ coordinate system. As shown in Fig. 2b, for an initial 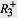 domain, the low-energy polarization switching path is within the 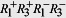 plane (*ϕ* = 0°). An electric field along [110] direction is required to overcome the energy barrier, which mainly results from the elastic and Landau-type bulk free energy, to switch the 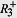 domain (*θ* = −58°, *ϕ* = 0°) to the metastable (see the saddle point in the energy density profile) 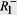 (*θ* = 70°, *ϕ* = 0°), which would further relax to the 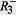 (*θ* = 112°, *ϕ* = 0°) domain. Although the electric energy increases as the head to tail 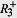
(unpoled region)/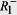
(poled region) 109°domain wall changes into the 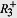/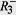 domain wall during the latter process, the elastic energy decreases more significantly. Indeed, when the polarization underneath the CoFe dot is 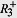, 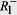 and 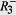, the corresponding elastic and electric energy densities (*f*_*elastic*_, *f*_*electric*_) are (6.91 MJ/m^3^, −4.01 MJ/m^3^), (14.1 MJ/m^3^, −4.18 MJ/m^3^), and (5.97 MJ/m^3^, 2.64 MJ/m^3^), respectively.

Figure 2c further shows the time-dependent evolution of the 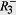 domain volume fraction in the BFO region underneath the CoFe electrode after removing the negative voltage of −2*V*_c_, with the in-plane size of the CoFe dot varying from 32 nm × 32 nm to 192 nm × 192 nm. Once removing the voltage, the 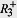/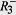
multi-domain will evolve back to the 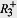 single-domain to reduce the electric energy, which is evidenced by the presence of global energy minima at the 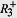 single-domain in the profile shown in Fig. 2b. Such relaxation of high-energy domain structure has been experimentally observed in BFO thin films^36^,^37^. Nevertheless, the stability of the 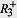/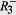 multi-domain can be improved by increasing the in-plane size of the CoFe electrode (e.g., to 192 nm × 192 nm), as shown in Fig. 2c.

**Figure 3:**
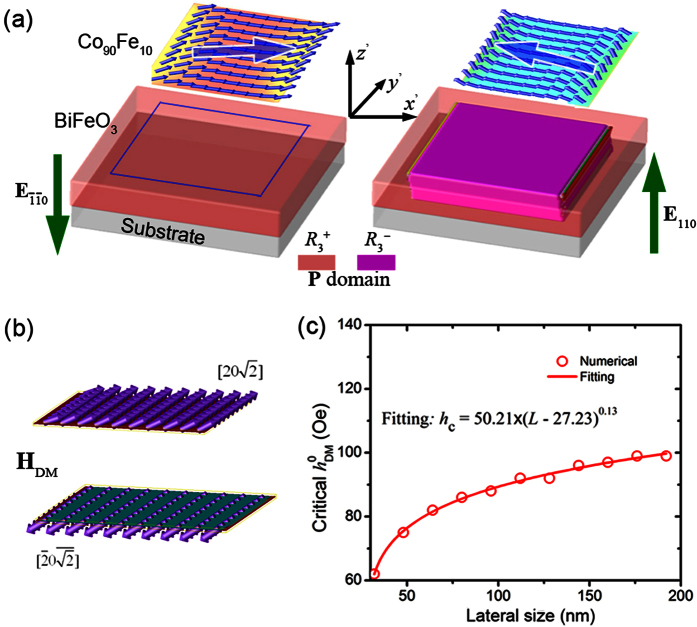
Voltage-controlled magnetism in CoFe/(110) BiFeO_3_ heterostructure. **** (**a**) Perpendicular electric field induced ferroelectric and ferromagnetic domains switching in dot CoFe/(110)-thin-film BFO multiferroic heterostructure mediated by (**b**) the interfacial DM fields. (**c**) Dependence of the critical DM field strength which can make the net magnetization switch 150° under perpendicular voltage on the in-plane lateral size (length and width) of CoFe dots.

Now turn to discuss how the perpendicular electric field modulates the magnetization distribution in CoFe dots through the interfacial exchange interaction mechanism. As discussed above, the (110) BFO thin film can be pre-poled to become a single domain over a micron scale range, for instance, the 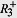 single domain obtained by applying a positive voltage along 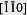 direction (see Fig. 3a). In this case, the magnetization distribution in the CoFe dot of 192 nm × 192 nm × 2.5 nm exhibits a typical ‘leaf’-like ground state structure^38^ with average magnetization component *m*_*x*’_ = 0.90, *m*_*y*’_ = 0.31 under the interface **H**_DM_ field along the 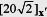 direction (see the top part of Fig. 3b). As the electric field reverses to be along [110] direction under negative voltages (on the right of Fig. 3a), the local 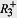 domain in the region underneath the CoFe dot transforms into a local 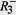 domain by successive 109° and 71° switching, leading to a reoriented interface **H**_DM_ field along the 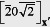 direction (see the bottom part of Fig. 3b). Accordingly, the average magnetization components gradually changes to *m*_*x*’_ = −0.90, *m*_*y*’_ = 0.31, i.e., it occurs a 150° reversal of the net magnetization along the in-plane *x*’ axis. Note that such reversal of in-plane net magnetization requires a sufficiently large magnitude of the interface **H**_DM_ field (namely, 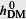). The critical value of 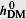 is dependent on the in-plane size of the CoFe dot. As shown in Fig. 3c, the critical 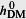 value increases with increasing in-plane lateral size *L* (the length and width being the same) as a power-function. Specifically, for the CoFe dots with in-plane size below 192 nm × 192 nm, the critical 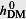 value is about 100 Oe, quite close to the experimentally measured value (about 100 Oe) in a similar layered heterostructure of polycrystalline CoFe thin film and (001) BFO thin films^15^.

**Figure 4:**
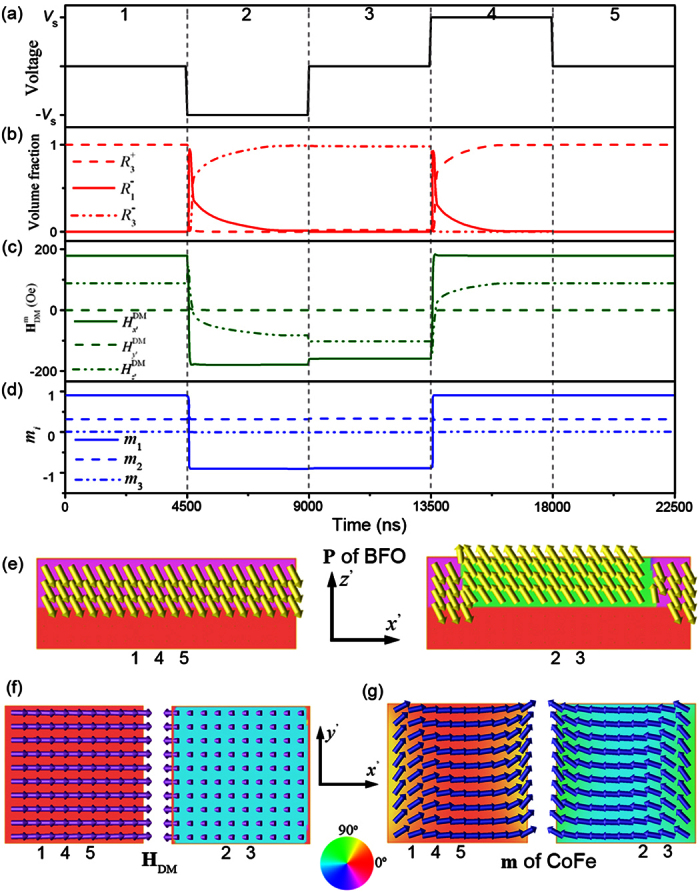
Polarization and magnetization dynamics in CoFe dot/(110)-BiFeO_3_ film under voltage. **** (**a**) Perpendicular voltage pulse applied on the poly-dot CoFe/(110)-thin-film BFO multiferroic heterostructure. (**b**) Voltage pulses induced ferroelectric phase evolutions of 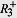, 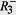, 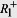, and 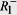 in BFO thin-film layer. (**c**) Voltage pulses induced interfacial **H**_DM_ field and hence (**d**) the magnetization evolution in CoFe dots. Voltage-induced corresponding (**e**) polarization domain-vector morphology in x’-z’ middle cross-sectional plane of BFO-substrate layers, (**f**) the 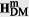, and (**g**) magnetization domain-vector morphologies in x’-y’ plane middle cross-sectional plane of CoFe layer, respectively. The color wheel indicates the **P** , 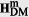, or **m** orientation in the their correspondingly exhibited planes.

Figure 4 further shows the evolution of the distributions of polarization, interface **H**_DM_ field, and magnetization under successive square-wave bipolar voltage pulses with a constant duration of 4500 ns (Fig. 4a) in the CoFe dot (192 nm × 192 nm × 2.5 nm)/(110) BFO heterostrucuture. As it can be seen, non-volatile behaviors are exhibited for the 180° ferroelectric reversal (Fig. 4b,e) and subsequent reversal of the interface **H**_DM_ field from 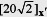 to 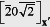 (see Fig. 4c,f). As a result, the in-plane magnetization component *m*_*x*’_ can be reversed back and forth between the bistable states of −0.9 and 0.9 (i.e., a 150° reversal, see Fig. 4d,g) under perpendicular voltage. Moreover, as the magnetization switching (~75 ns) in the CoFe dot is much faster than the ferroelectric switching (~4500 ns) in the BFO thin film, the ferroelectric switching is the time-determining step during such perpendicular voltage-induced magnetization reversal.

**Figure 5:**
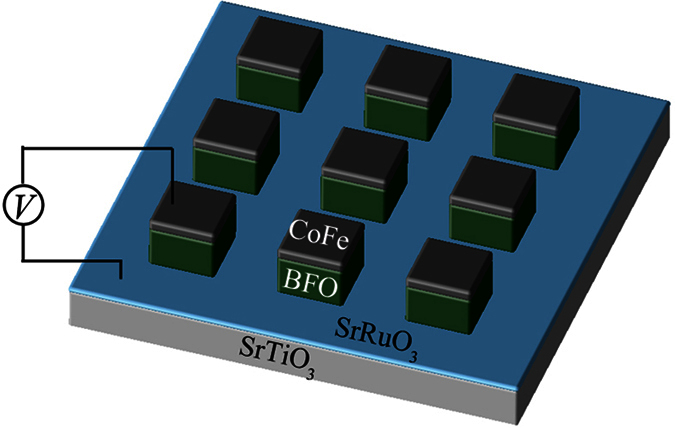
Patterned CoFe/BiFeO_3_ island heterostructure. **** Dot CoFe/(110)-island BFO multiferroic heterostructures grown on (110) SrTiO_3_ substrate with CoFe and SrRuO_3_ as the top and bottom electrodes, respectively.

**Figure 6:**
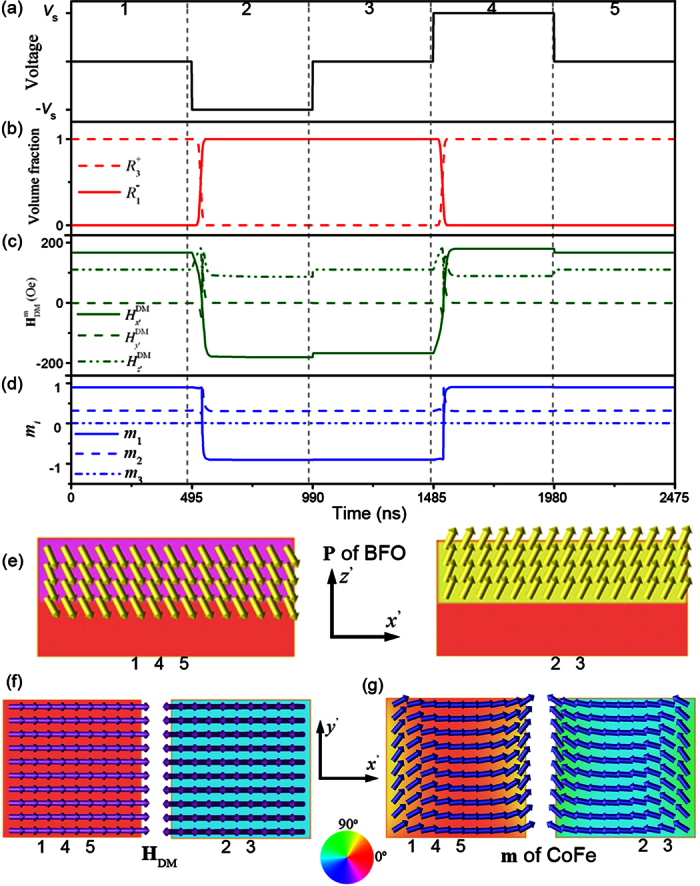
Polarization and magnetization dynamics in CoFe dot/(110)-BiFeO_3_ island under voltage. **** (**a**) Perpendicular voltage pulse applied on the poly-dot CoFe/(110)-island BFO multiferroic heterostructure. (**b**) Voltage pulses induced ferroelectric phase evolutions of 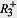 and 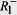 in BFO island layer. (**c**) Voltage pulses induced interfacial **H**_DM_ field and hence (**d**) the magnetization evolution in CoFe dots. Voltage-induced corresponding (**e**) polarization domain-vector morphology in x’-z’ middle cross-sectional plane of BFO-substrate layers, (**f**) the 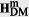, and (**g**) magnetization domain-vector morphologies in x’-y’ plane middle cross-sectional plane of CoFe layer, respectively. The color wheel indicates the **P** , 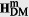, or **m** orientation in the their correspondingly exhibited planes.

The entire time of reversal, however, can be reduced by cutting out the continuous (110) BFO thin film into isolated islands so that the overlaying CoFe dot covers the whole surface of BFO (see Fig. 5). In this case, the polarization time can be greatly reduced because of the significantly released elastic energy. As demonstrated in Fig. 6, upon a negative voltage pulse of 495 ns (see the second stage of Fig. 6a), a single-domain 109° polarization switching from 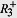 to 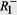 happens (Fig. 6b,e), leading to a reversal of the in-plane component of interface **H**_DM_ field from 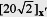 to 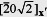 (Fig. 6c,f). A similar 150° reversal of in-plane net magnetization occurs accordingly (Fig. 6d,g). Note that the time of 109° polarization switching is about 45 ns, which is the same as the magnetization switching time of 45 ns. As a result, the overall magnetization switching time driven by the perpendicular voltage is also 45 ns, which is about 100 times faster than the case in the heterostructures involving BiFeO_3_ thin films (i.e., about 4500 ns as shown in Fig. 4).

**Figure 7:**
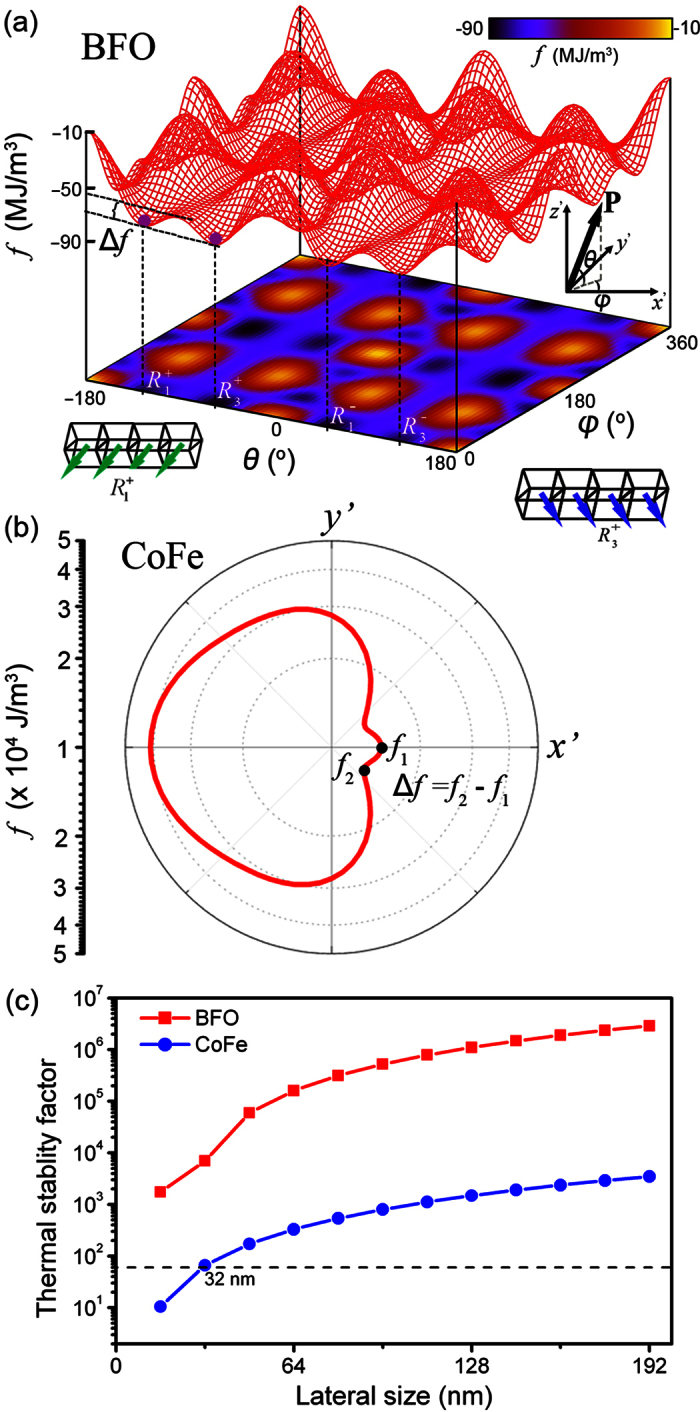
Thermal stability analysis in CoFe dot/(110)-BiFeO_3_ island. **** (**a**) Energy profile as function of polarization orientation for single-domain BFO islands. (**b**) Energy polar plot for an in-plane magnetized CoFe dot with a size of 192 nm × 192nm × 192 nm when the polarization in the bottom single-domain BFO island is along the 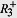 direction. (**c**) Thermal stability factors as function of the respective lateral sizes (length and width) of BFO and CoFe. The thermal stability can be obtained from the energy profile by extracting the barrier (Δ*f*) between the adjacent energy minimal.

Another advantage of using BiFeO_3_ island based heterostructure is the significantly improved non-volatility (and thermal stability). Unlike the case of a continuous BFO thin film where the 180° domain wall between the poled region (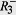) and the unpoled region (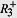) leads to high electric energy, the 109° switching in the present single-domain BFO island (Fig. 6b) is thermodynamically stable. Figure 7a shows the total free energy density profile of a single-domain BFO island by assuming the polarization pointing along every direction in the *x*’*y*’*z*’ coordinate system. As it can be seen, 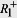, 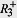, 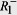, and 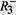 are thermodynamically degenerate with equal depth at equilibrium states. The barrier (Δ*f*) between 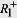 and 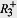 or 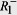 and 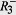 determines the stability of polarization under thermal fluctuation. For the CoFe dot only with in-plane magnetization, the energy barrier Δ*f* between the global energy minima at *f*_2_ and the metastable state *f*_1_ (induced by the interface **H**_DM_ field along the 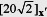 direction) determines the thermal stability of magnetization, as shown in Fig. 7b. Accordingly, the thermal stability factors of BFO island and CoFe dot can be calculated as 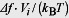, where *k*_B_ and *T* are the Boltzmann constant and temperature in Kelvin, respectively, and *V*_*i*_ (*i* = BFO, CoFe) is the volume.

Figure 7c further shows the thermal stability factor of both BFO islands and CoFe dots as a function of their in-plane sizes, with their thicknesses fixed at 24 nm and 2.5 nm, respectively. As seen, the stability factors of the BFO islands are at least two orders of magnitude higher than those of the CoFe dots, indicating that the thermal stability of the heterostructure is determined by the latter. Such high thermal stability of polarization in single-domain BFO islands results from the high potential barrier between the degenerate polarization states from the Landau-type bulk free energy. Nevertheless, the thermal stability factor of the CoFe dot can still be larger than 60 as its in-plane size exceeds 32 nm × 32 nm, suggesting a long timescale retention of magnetization states up to 10 years in the ideal case^39^,^40^.

## Discussion

In summary, perpendicular voltage-driven reversal of in-plane magnetization reversal has been demonstrated by phase-field simulations in multiferroic magnetoelectric heterostructures composed of polycrystalline CoFe dots and (110) BiFiO_3_ continuous film or island. In the clamped BiFeO_3_ thin film, the 180° ferroelectric reversal occurs by successive 109°and 71° ferroelastic switching. The non-volatility of such ferroelectric reversal can be enhanced by increasing the in-plane size of the overlaying CoFe dot, to alleviate the energy competition between the poled region underneath the CoFe dot and the rest region. Associated with repeatable polarization reversal, a repeatable 150° reversal of in-plane net magnetization reversal in the CoFe dot has been further demonstrated due to the reversal of the in-plane component of the interface **H**_DM_ field.

Similar non-volatile and repeatable voltage-induced magnetization reversal has been demonstrated when the BiFiO_3_ thin film is etched into islands to release the substrate clamping and to eliminate the competition between the poled and unpoled regions. In such CoFe dot/BiFeO_3_ island heterostructure, bistable 109° ferroelastic switching in single-domain BiFeO_3_ has been demonstrated, which leads to 100 times faster ferroelectric switching (and hence faster overall response). As the switching time is estimated according to the Kolmogorov–Avrami–Ishibashi model (see Methods) which assumes that the polarization reversal occurs by domain wall nucleation and propagation, the calculated time (Fig. 6) for uniform switching in the CoFe dot/BiFeO_3_ island heterostructure could be overestimated, i.e., the actual switching speed could be faster in the CoFe dot/BiFeO_3_ island heterostructure, since we used the same value of the kinetic coefficient *L* in the TDGL equation for the BiFeO_3_ island as for the BiFeO_3_ thin film due to unknown for the island. Actually, the *L* values are different for continuous thin film and isolated island due to their different strain conditions, and should be larger in the BiFeO_3_ island than in the clamped continuous thin film. A larger *L* in the BiFeO_3_ island would yield a higher switching speed, though it is hard to estimate the actual switching speed in the island at present. Furthermore, the island heterostructure also shows good thermal stability even when the in-plane size of the heterostructure decreases down to 32 nm × 32 nm. These predictions would provide further directions for experimental studies of the BiFeO_3_-based multiferroic heterostructures for potential spintronic device applications.

## Methods

### Phase-field model

In phase field modeling of the magnetic/BFO multiferroic heterostructures, the spatial distributions of local polarization and magnetization vectors are used to describe the ferroelectric and ferromagnetic domain structures, respectively. As the ferroelectric phase of BiFeO_3_ has a rhombohedrally distorted perovskite structure with space group *R*3*c*, the spontaneous polarization of BiFeO_3_ is along the pseudocubic <111>_c_ in coordinate system *xyz* with *x*, *y*, and *z* along the [100]_c_, [010]_c_, and [001]_c_ directions, respectively, giving rise to the formation of eight possible polarization variants, i.e., 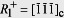, 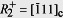, 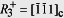, 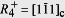, 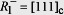 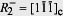, 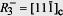, 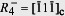
(see Fig. 1). For studying the ferroelectric domain structure in (110) BFO thin films, we introduce another coordinate system *x*’*y*’*z*’ with *x*’, *y*’, *z*’ along the [001], 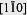, and [110] directions (see Fig. 1a). The polarization vector 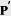 in the *x*’*y*’*z*’ coordinate system is chosen to be evolved by the time-dependent Landau-Ginzburg (TDGL) equation^41^, i.e.,


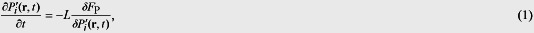


where *L* the kinetic coefficient related to the domain wall mobility and *F*_P_ the total free energy of the FE layer, respectively. The total free energy of the FE layer includes the bulk, elastic, electric, and the gradient energies, i.e.,


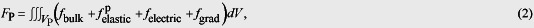


where *V*_P_ represents the volume of the FE layer in the heterostructure.

The expressions for the bulk, elastic, electric, and gradient energy densities were used as before^41^,^42^,^43^. The correspondence of the polarization components *P*_*j*_ in coordinate system *xyz* to the counterparts 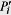 in *x*’*y*’*z*’ is 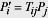 with *T*_*ij*_ the transformation matrix given as follows:


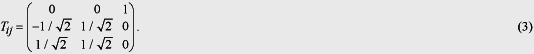


Note that when calculating the elastic, electric, and gradient energy densities, the related tensors including the electrostrictive coefficient tensor, background dielectric constant tensor, gradient energy coefficient tensor have to be performed transformation from coordinate system *xyz* to the *x*’*y*’*z*’ (see Ref. 41). For solving the mechanical equilibrium equation, Khachaturyan’s microelastic theory^44^ is employed by incorporating the thin-film^45^ or the isolated-island^43^,^46^ boundary conditions for the cases of BFO continuous film and island, respectively. For (110) BFO film grown on (110) SrTiO_3_ substrate under full constraint, the film/substrate mismatch strain 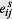 are 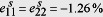 and 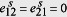
47,48.

For solving the electrostatic equilibrium equation, the Fast Fourier Transformation method is employed by incorporating the short-circuit surface boundary condition^49^ through which the externally applied voltage on BFO by CoFe and SrRuO_3_ bottom electrode is introduced into the phase field model.

For determining the real time scale corresponding to each iteration step of the TDGL equation, the switching dynamics as a function of iteration step is obtained and compared to the Kolmogorov–Avrami–Ishibashi (KAI) model^50^,^51^ as following


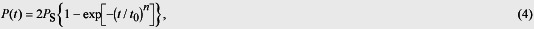


where *P*(*t*) the switched polarization, *t* the real time, *P*_S_ the saturated polarization, *t*_0_ the characteristic switching time, and *n* the effective dimension of domain growth. For (110) BFO thin film, *n* has a value of 2 and *t*_0_ is about 1.5 × 10^−7^s with the applied electric field of 250 kV/cm^34^. Our simulation shows that a real time interval of Δ*t* = 8.25 × 10^−10^s for the polarization domain evolution is determined corresponding to the each iteration step of TDGL equation.

The evolution of the magnetic domain structures of the FM layer can be described by the Landau-Lifshitz-Gilbert (LLG) equation, i.e.,


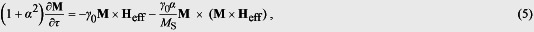


where *γ*_0_ the gyromagnetic ratio and α the Gilbert damping constant, respectively. The real time interval for the magnetic domain evolution determined by Δτ(1+α^2^)/(*γ*_0_*M*_s_) corresponding to each iteration step of Eq. (5) is about 0.06 ps with 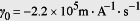 (from Ref. 52), α = 0.01 (from Ref. 32), and Δτ = 0.02. **H**_eff_ is the effective magnetic field, given as 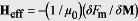, with *μ*_0_ denoting the vacuum permeability and *F*_m_ the total free energy of the CoFe layer. The *F*_m_ is formulated as,


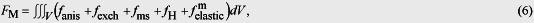


where *f*_anis_, *f*_*exch*_, *f*_*ms*_, *f*_*H*_, and 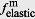 the magnetocrystalline anisotropy, exchange, magnetostatic, the **H**_DM_-field, and elastic energy densities, respectively. Regarding that the CoFe layer is grown in isotropically polycrystalline or amorphous states, all the above energy density expressions are independent of the coordinate system. Among them, the *f*_anis_ is neglected for simplicity due to the isotropic nature of the polycrystalline film. The isotropic *f*_exch_ is determined by the gradient of local magnetization vectors, i.e.,


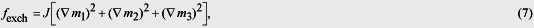


where *J* denotes the exchange stiffness constant. The magnetostatic energy density *f*_ms_ can be written as,


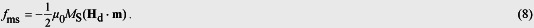


Here **H**_**d**_denotes the stray field, and it can be numerically calculated by employing a finite-size magnetostatic boundary condition previously developed for a 3D array of ferromagnetic cubes^53^.

For the **H**_DM_-field induced energy density, it is similar to the Zeeman energy of an external magnetic field, and can be expressed as


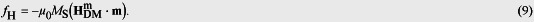


Here the 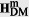 indicates the **H**_DM_-field that penetrates onto the CoFe film through the interface, and is given by,


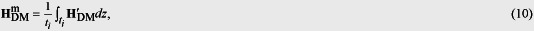


where *t*_*i*_ denotes the thickness of the interface creating interfacial magnetic interaction, and the 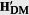 represents the **H**_DM_-field vector in the BFO layer in coordinate system *x*’*y*’*z*’. 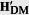 can be obtained from **H**_DM_ using coordinate transformation 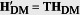, wherein the **H**_DM_ can be obtained through **H**_DM_ = **P** × **L**. With regard to the antiferromagnetic vector **L**, it was predicted with a sixfold degeneracy in bulk BiFeO_3_ system. However, in thin-film system, this sixfold degeneracy is broken and only one easy axis remains, according to the experimental observations and first-principle calculations^33^. Therefore, the preserved antiferromagnetic vector **L** coinciding with **P** for thin-film BiFeO_3_ in *xyz* coordinate system can be phenomenologically described by


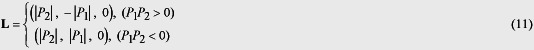


or the correspondingly equivalent directions. From Eq. (11) one can see that the antiferromagnetic vector **L** remains for the perpendicular 71° and 180° polarization switching under perpendicular voltage, while it rotates 90° for the in-plane 71° polarization switching under in-plane voltage in (001) BiFeO_3_ thin films, in consistent with the experimental results^13^,^33^. Thus, the DM effect induced **H**_DM_ field can be written as


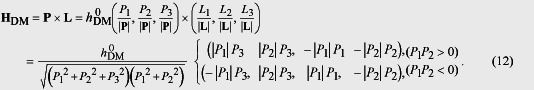


The *P*_*i*_ and *L*_*i*_ (*i* = 1,2,3) are the components of the polarization vectors and the antiferromagnetic vectors in coordinate system *xyz*, and the 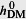 represents the magnitude of the **H**_DM_ field. Note that the expression of **H**_DM_ field published in our previous paper is just a specific case for studying the in-plane 71° polarization switching in (001) BiFeO_3_ thin film^13^. Equation (12) clearly indicates that **P**-dependent nature of the **H**_DM_ field related to individual ferroelectric domain at the BFO surface, which can propagate across the hetero-interface and act on the CoFe dots. Combining Eqs. (3) and (12), Eq. (10) can be rewritten as,


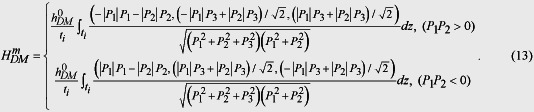


The formulation of the elastic energy density 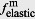 of CoFe layer is also same as before^16^. Note that for a (110) BFO film under perpendicular voltage approaching to saturation the in-plane ferroelastic strain remains unchanged whenever the polarization vector is up or down, thus only the structural strain introduced during growth of CoFe is affecting the initial magnetization.

Temporal evolutions of the ferroelectric and magnetic domain structures are obtained by numerically solving the TDGL and LLG equations using semi-implicit Fourier spectral method and Gauss-Seidel projection method, respectively. The material parameters used for simulations, including the Landau coefficients, electrostrictive coefficients, elastic constants of BFO layer, and the saturated magnetization, exchange stiffness constant, elastic constants of CoFe layer can be found in the literature^16^,^41^,^54^ and are listed as following: 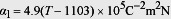 (where the temperature *T* is in K), 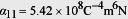, 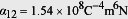, 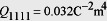, 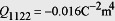, 
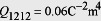
, 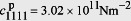, 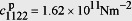, 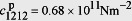; For isotropically amorphous CoFe film, 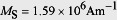, 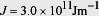, 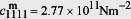, 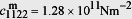, 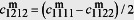. The discrete grid points of 128Δ*x* × 128Δ*y* × 48Δ*z* with real grid space Δ*x* = Δ*y* = Δ*z* = 1 nm are employed to describe the BFO film/substrate system, wherein the thickness of BFO *t*_p_ is taken as 24 nm by setting *t*_p_ = 24Δ*z*, and the thickness of the interface *t*_i_ creating the interfacial magnetic interaction is assigned a value of 4 nm by setting *t*_i_ = 4Δ*z*. While for CoFe dots, discrete grid points of *N*Δ*x* × *N*Δ*y* × 20Δ*z* with Δ*x* = Δ*y* = 1 nm, and Δ*z* = 0.5 nm are used, where the thickness of the CoFe *t*_m_ is set to be 2.5 nm by taking *t*_m_ = 5Δ*z* and the in-plane size *N*Δ*x* can be given by setting *N*. When the in-plane size of CoFe layer is larger than 96 × 96 nm^2^, the real size of the heterostructure is achieved by changing Δ*x* and Δ*y* but keeping *N* = 96. For the BFO island, the discrete grid points of 128Δ*x* × 128Δ*y* × 48Δ*z* with real grid space Δ*x* = Δ*y* = 2 nm and Δ*z* = 1 nm are employed with *N*Δ*x* × *N*Δ*y* × 24Δ*z* of them are occupied by the BFO island while the rest are the air which allows the lateral relaxation of the island.

## Additional Information

**How to cite this article**: Wang, J. J. *et al.* Magnetization Reversal by Out-of-plane Voltage in BiFeO_3_-based Multiferroic Heterostructures. *Sci. Rep.* **5**, 10459; doi: 10.1038/srep10459 (2015).

## References

[b1] Eerenstein W., Mathur N. D. & Scott J. F. Multiferroic and magnetoelectric materials. Nature 442, 759 (2006). 16915279 10.1038/nature05023

[b2] Duan C. G., Jaswal S. S. & Tsymbal E. Y. Predicted magnetoelectric effect in Fe/BaTiO_3_ multilayers: ferroelectric control of magnetism. Phys. Rev. Lett. 97, 047201 (2006). 16907608 10.1103/PhysRevLett.97.047201

[b3] Vaz C. A. F. Electric field control of magnetism in multiferroic heterostructures. J. Phys: Condens. Matter 24, 333201 (2012). 22824827 10.1088/0953-8984/24/33/333201

[b4] Cuellar F. A. *et al.* Reversible electric-field control of magnetization at oxide interfaces. Nature Commun. 5, 4215 (2014). 24953219 10.1038/ncomms5215

[b5] Garcia V. *et al.* Ferroelectric control of spin polarization. Science 327, 1106 (2010). 20075211 10.1126/science.1184028

[b6] Ramesh R. & Spaldin N. Multiferroics: progress and prospects in thin films. Nature Mater. 6, 21 (2007). 17199122 10.1038/nmat1805

[b7] Vaz C. A. F., Hoffman J., Ahn C. H. & Ramesh R. Magnetoelectric coupling effects in multiferroic complex oxide composite structures. Adv. Mater. 22, 2900 (2010). 20414887 10.1002/adma.200904326

[b8] Ma J., Hu J. M., Li Z. & Nan C. W. Recent progress in multiferroic magnetoelectric composites: from bulk to thin films. Adv. Mater. 23, 1062 (2011). 21294169 10.1002/adma.201003636

[b9] Chu Y. H., Martin L. W., Holcomb M. B. & Ramesh R. Controlling magnetism with multiferroics. Mater. Today 10, 16 (2007).

[b10] Chu Y.-H. *et al.* Electric-field control of local ferromagnetism using a magnetoelectric multiferroic. Nat. Mater. 7, 478–482 (2008). 18438412 10.1038/nmat2184

[b11] Lebeugle D., Mougin A., Viret M., Colson D. & Ranno L. Electric field switching of the magnetic anisotropy of a ferromagnetic layer exchange coupled to the multiferroic compound BiFeO_3_. Phys. Rev. Lett. 103, 257601 (2009). 20366284 10.1103/PhysRevLett.103.257601

[b12] Wu S. M. *et al.* Reversible electric control of exchange bias in a multiferroic field-effect device. Nat. Mater. 9, 756 (2010). 20657590 10.1038/nmat2803

[b13] Heron J. *et al.* Electric-field-induced magnetization reversal in a ferromagnet-multiferroic heterostructure. Phys. Rev. Lett. 107, 217202 (2011). 22181917 10.1103/PhysRevLett.107.217202

[b14] Huijben M., Yu P., Martin L. W., Molegraaf H. J., Chu Y. H., Holcomb M. B., Balke N., Rijnders G. & Ramesh R. Ultrathin limit of exchange bias coupling at oxide multiferroic/ferromagnetic interfaces Adv. Mater. 25, 4739 (2013). 23847010 10.1002/adma.201300940

[b15] Trassin M. *et al.* Interfacial coupling in multiferroic/ferromagnet heterostructures. Phys. Rev. B 87, 134426 (2013).

[b16] Wang J. J., Hu J.M., Yang T. N., Feng M., Zhang J. X., Chen L. Q. & Nan C.W. Effect of strain on voltage-controlled magnetism in BiFeO_3_-based heterostructures. Sci. Rep. 4, 4553 (2014). 24686503 10.1038/srep04553 PMC3971450

[b17] Hambe M., Petraru A., Pertsev N. A., Munroe P., Nagarajan V. & Kohlstedt H. Crossing an Interface: Ferroelectric control of tunnel currents in magnetic complex oxide heterostructures. Adv. Funct. Mater. 20, 2436 (2010).

[b18] Rana A., Lu H., Bogle D. K., Zhang Q., Vasudevan R., Thakare V., Gruverman A., Ogale S. & Nagarajan V. Scaling behavior of resistive switching in epitaxial bismuth ferrite heterostructures. Adv. Funct. Mater. 24, 3962 (2014).

[b19] Hu J. M., Li Z., Chen L.Q. & Nan C. W. High-density magnetoresistive random access memory operating at ultralow voltage at room temperature. Nature Commun. 2, 553 (2011) 22109527 10.1038/ncomms1564 PMC3482632

[b20] Hu J. M., Li Z., Chen L.Q. & Nan C.W. Design of a voltage-controlled magnetic random access memory based on anisotropic magnetoresistance in a single magnetic layer. Adv. Mater. 24, 2869 (2012). 22544814 10.1002/adma.201201004

[b21] Heron J. *et al.* Deterministic switching of ferromagnetism at room temperature using an electric field. Nature 516, 370 (2014). 25519134 10.1038/nature14004

[b22] Zhang J. X., Li Y. L., Schlom D. G., Chen L. Q., Zavaliche F., Ramesh R. & Jia Q. X. Phase-field model for epitaxial ferroelectric and magnetic nanocomposite thin film. Appl. Phys. Lett. 90, 052909 (2007).

[b23] Hu J. M., Yang T., Chen L. & Nan C. W. Voltage-driven perpendicular magnetic domain switching in multiferroic nanoislands. J. Appl. Phys. 113, 194301 (2013).

[b24] Cruz M. P., Chu Y. H., Zhang J. X., Yang P. L., Zavaliche F., He Q., Shafer P., Chen L. Q. & Ramesh R. Strain control of domain-wall stability in epitaxial BiFeO_3_ (110) films. Phys. Rev. Lett. 99, 217601 (2007). 18233258 10.1103/PhysRevLett.99.217601

[b25] Tseng A. A. Recent developments in nanofabrication using ion projection lithography. Small 1, 594 (2005). 17193492 10.1002/smll.200500050

[b26] Chu Y. H., Cruz M. P., Yang C. H., Martin L. W., Yang P. L., Zhang J. X., Lee K., Yu P., Chen L. Q. & Ramesh R. Domain control in multiferroic BiFeO_3_ through substrate vicinality Adv. Mater. 19, 2662 (2007).

[b27] Chu Y. H. *et al.* Nanoscale domain control in multiferroic BiFeO_3_ thin films Adv. Mater. 18, 2307 (2006).

[b28] Dzyaloshinskii I. E. Thermodynamic theory of ‘weak’ ferromagnetism in antiferromagnetic substances. Sov. Phys. JETP 5, 1259 (1957).

[b29] Moriya T. Anisotropic superexchange interaction and weak ferromagnetism Phys. Rev. 120, 91 (1960).

[b30] Ederer C. & Spaldin N. A. Weak ferromagnetism and magnetoelectric coupling in bismuth ferrite. Phys. Rev. B 71, 060401 (2005).

[b31] Ederer C. & Fennie C. J. Electric-field switchable magnetization via the Dzyaloshinskii–Moriya interaction: FeTiO_3_ versus BiFeO_3_. J. Phys.: Condens. Matter 20, 434219 (2008).

[b32] Qiu D., Ashraf K. & Salahuddin S. Nature of magnetic domains in an exchange coupled BiFeO_3_/CoFe heterostructure. Appl. Phys. Lett. 102, 112902 (2013).

[b33] Zhao T., *et al.* Electrical control of antiferromagnetic domains inmultiferroic BiFeO_3_ films at room temperature Nature Mater. 5, 823(2006). 16951676 10.1038/nmat1731

[b34] Pantel D., Chu Y. H., Martin L. W., Ramesh R., Hesse D. & Alexe M. Switching kinetics in epitaxial BiFeO_3_ thin films. J. Appl. Phys. 107, 084111(2010).

[b35] Gruverman A., Wu D. & Scott J. F. Piezoresponse force microscopy studies of switching behavior of ferroelectric capacitors on a 100-ns time scale. Phys. Rev. Lett. 100, 097601(2008). 18352748 10.1103/PhysRevLett.100.097601

[b36] Baek S. H. *et al.* Ferroelastic switching for nanoscale non-volatile magnetoelectric devices Nature Mater. 9, 309(2010). 20190772 10.1038/nmat2703

[b37] Nelson C. T. *et al.* Domain dynamics during ferroelectric switching. Science 334, 968(2011). 22096196 10.1126/science.1206980

[b38] Cowburn R. P. & Welland M. E. Micromagnetics of the single-domain state of square ferromagnetic nanostructures. Phys. Rev. B 58, 9217 (1998).

[b39] Zhu J. G. Magnetoresistive random access memory: the path to competitiveness and scalability. Proceedings of the IEEE 96, 1786 (2008).

[b40] Evans R., Chantrell R.W., Nowak U., Lyberatos A. & Richter H. J. Thermally induced error: Density limit formagnetic data storage. Appl. Phys. Lett. 100, 102402 (2012).

[b41] Zhang J. X., Li Y. L., Choudhury S., Chen L. Q., Chu Y. H., Zavaliche F., Cruz M. P., Ramesh R. & Jia Q. X. Computer simulation of ferroelectric domain structures in epitaxial BiFeO_3_ thin film. J. Appl. Phys. 103, 094111 (2008).

[b42] Wang J. J., Wu P. P., Ma X. Q. & Chen L. Q. Temperature-pressure phase diagram and ferroelectric properties of BaTiO_3_ single crystal based on a modified Landau potential. J. Appl. Phys. 108, 114105 (2010).

[b43] Wang J. J., Ma X. Q., Li Q., Britson J. & Chen L. Q. Phase transitions and domain structures of ferroelectric nanoparticles: Phase field model incorporating strong elastic and dielectric inhomogeneity. Acta Mater. 61, 7591 (2013).

[b44] Khachaturyan A. Theory of structural transformations in solid. (Wiley, 1983).

[b45] Li Y. L., Hu S. Y., Liu Z. K. & Chen L. Q. Effect of substrate constraint on the stability and evolution of ferroelectric domain structures in thin film. Acta Mater. 50, 395 (2002).

[b46] Zhang J. X., Wu R., Choudhury S., Li Y. L., Hu S. Y. & Chen L. Q. Three-dimensional phase-field simulation of domain structures in ferroelectric islands. Appl. Phys. Lett. 92, 122906 (2008).

[b47] Zavaliche F., Yang S. Y., Zhao T., Chu Y. H., Cruz M. P., Eom C. B. & Ramesh R. Multiferroic BiFeO_3_ films: domain structure and polarization dynamics. Phase Transitions 79, 991 (2006).

[b48] Schlom D. G., Chen L. Q., Eom C. B., Rabe K. M., Streiffer S. K. & Triscone J. M. Strain tuning of ferroelectric thin films. Annu. Rev. Mater. Res. 37, 589 (2007).

[b49] Li Y. L., Hu S. Y., Liu Z. K. & Chen L. Q. Effect of electrical boundary conditions on ferroelectric domain structures in thin film. Appl. Phys. Lett. 81, 427 (2002).

[b50] Ishibashi Y. & Takagi Y. Note on ferroelectric domain switching. J. Phys. Soc. Jpn. 31, 506 (1971).

[b51] Ishibashi Y. Polarization reversal kinetics in ferroelectric liquid crystals. Jpn. J. Appl. Phys. Part 1 24,126 (1985).

[b52] Walowski J., Kaufmann M. D., Lenk B., Hamann C., McCord J. & Münzenberg M. Intrinsic and non-local Gilbert damping in polycrystalline nickel studied by Ti : sapphire laser fs spectroscopy. J. Phys. D: Appl. Phys. 41,164016 (2008).

[b53] Schabes M. E. & Aharoni A. Magnetostatic interaction fields for a three-dimensional array of ferromagnetic cubes. IEEE Trans. Magn. 23, 3882 (1987).

[b54] Hall R. C. Magnetic anisotropy and magnetostriction of ordered and disordered cobalt-iron alloys. J. Appl. Phys. 31, S157 (1960).

