# Corrigendum: Magnetization Reversal by Out-of-plane Voltage in BiFeO_3_-based Multiferroic Heterostructures

**DOI:** 10.1038/srep21443

**Published:** 2016-02-23

**Authors:** J. J. Wang, J. M. Hu, Ren-Ci Peng, Y. Gao, Y. Shen, L. Q. Chen, C. W. Nan

Scientific Reports 5: Article number:1045910.1038/srep10459; published online: 05212015; updated: 02232016

This Article contains a typographical error in a grant number in the Acknowledgements section.

“This work was supported by the NSF of China (Grant Nos 11234005, 51332001, 51221291, and 51472140), and the NSF (Grant No: DMR-1410714, DMR-0820404, and DMR-1210588).”

should read:

“This work was supported by the NSF of China (Grant Nos. 11234005, 51332001, 51221291, and 51472140), and the NSF (Grant No: DMR-1410714, DMR1420620, and DMR-1210588).”

